# Supplementary material for: Subcutaneously Injectable Hyaluronic Acid Hydrogel for Sustained Release of Donepezil with Reduced Initial Burst Release: Effect of Hybridization of Microstructured Lipid Carriers and Albumin
Source: Pharmaceutics. 2021 Jun 11;13(6):864. doi: 10.3390/pharmaceutics13060864 (PMC8230846; doi:10.3390/pharmaceutics13060864)
Supplement: Supplementary file 1 [file pharmaceutics-13-00864-s001.zip › pharmaceutics-1251959-supplementary.pdf]

pharmaceutics

Supplementary Materials: Subcutaneously Injectable Hyaluronic Acid Hydrogel for Sustained Release of Donepezil with Reduced Initial Burst Release: Effect of Hybridization of Microstructured Lipid Carriers and Albumin

Nae-Won Kang [1,†], So-Yeon Yoon [1,†], Sungho Kim [1], Na-Young Yu [1], Ju-Hwan Park [1,2], Jae-Young Lee [3], Hyun-Jong Cho [4] and Dae-Duk Kim [1,*]

Table S1. Solubility of donepezil in various liquid lipids.

Oils	Solubility (mg/mL)
**Oleic acid**	**183.51**
Miglyol 812	35.15
Olive oil	21.85
Peanut oil	18.74
Soy lecithin	8.80
Mineral oil	2.70

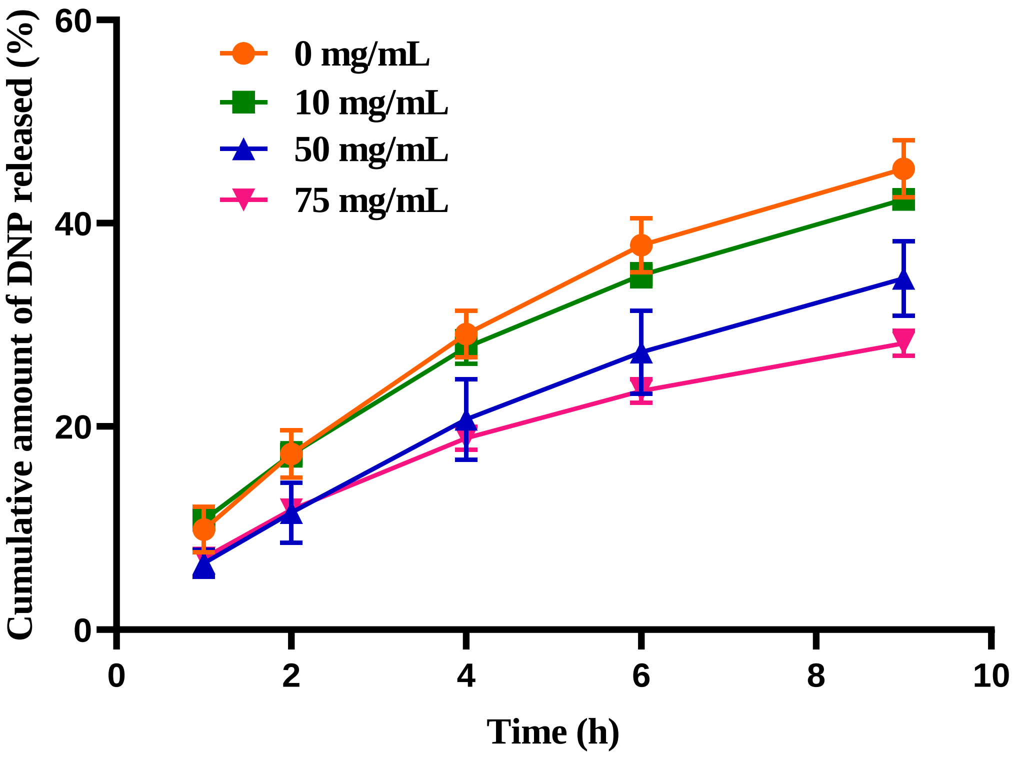

Figure S1. Effect of HSA amount (mg/mL) on the initial in vitro release of DNP from the MLC/HSA hydrogel. Different amounts of HSA (0–75 mg/mL) were added during the preparation of the MLC/HSA hydrogel. Data of "0 mg/mL" and "50 mg/mL" groups are the same as those for the "MLC hydrogel" and the "MLC/HSA hydrogel", respectively, in Figure 5A. HSA was suspended in "75 mg/mL" group. Each gel was loaded in the dialysis membrane (12–14 kDa molecular cut-off) sac for the release study. Each point represents the mean ± SD (*n* = 4).

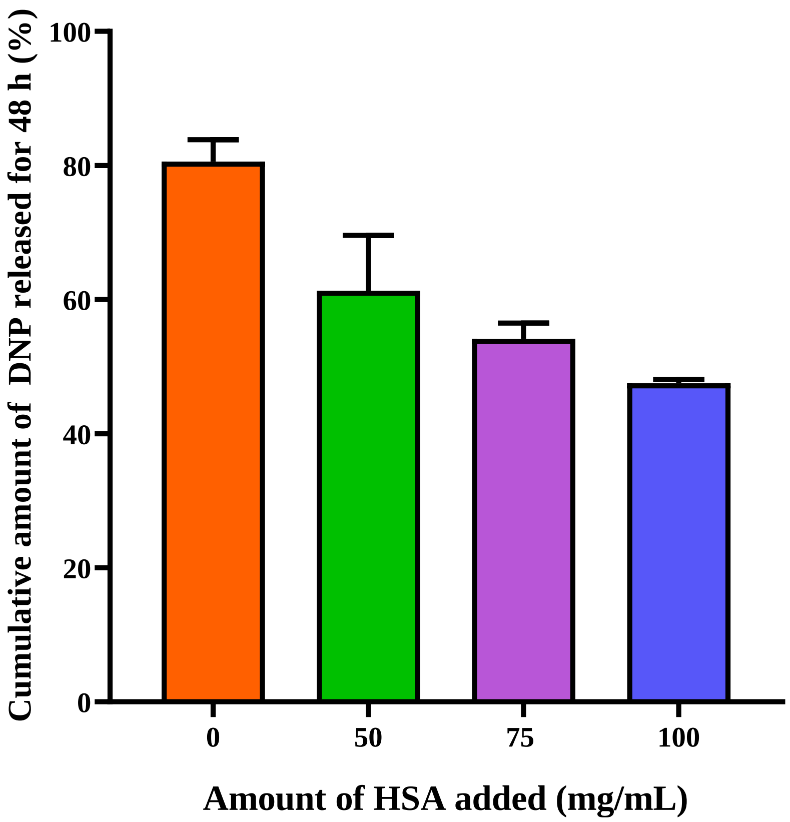

Figure S2. Effect of HSA amount (mg/mL) on the cumulative amount of DNP released for 48 h from the MLC/HSA hydrogel. Different amounts of HSA (0–100 mg/mL) were added during the preparation of the MLC/HSA hydrogel. Data of "0 mg/mL" and "50 mg/mL" groups are the same as those for the "MLC hydrogel" and the "MLC/HSA hydrogel", respectively, in Figure 5A. HSA was suspended in "75 mg/mL" and "100 mg/mL" groups. Each gel was loaded in the dialysis membrane (12–14 kDa molecular cut-off) sac for the release study. Each point represents the mean ± SD (n = 4).

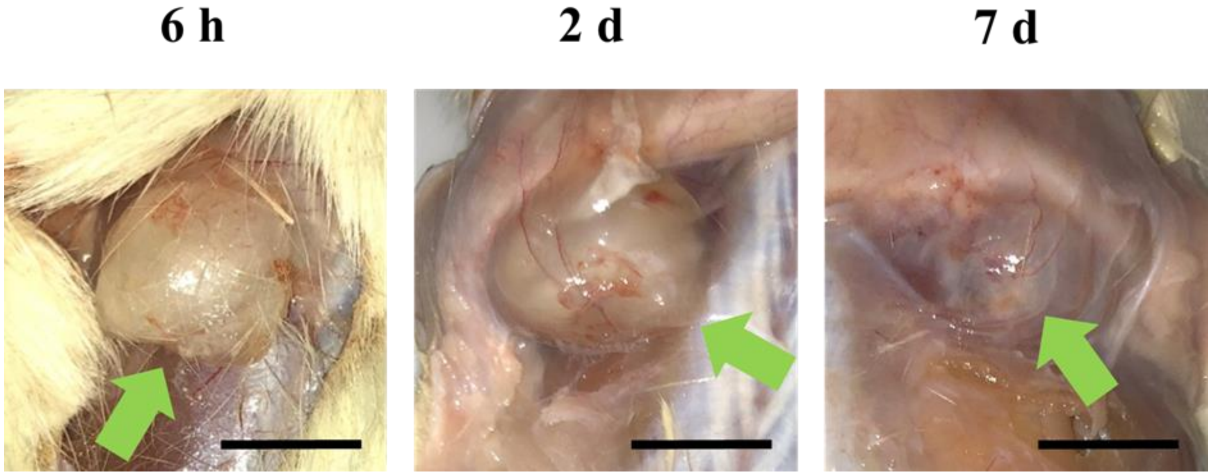

Figure S3. Optical images of subcutaneous tissues near the MLC/HSA hydrogel 6 h, 2 d, and 7 d after injection. Green arrows indicate the MLC/HSA hydrogel. The length of the scale bar is 2 cm.

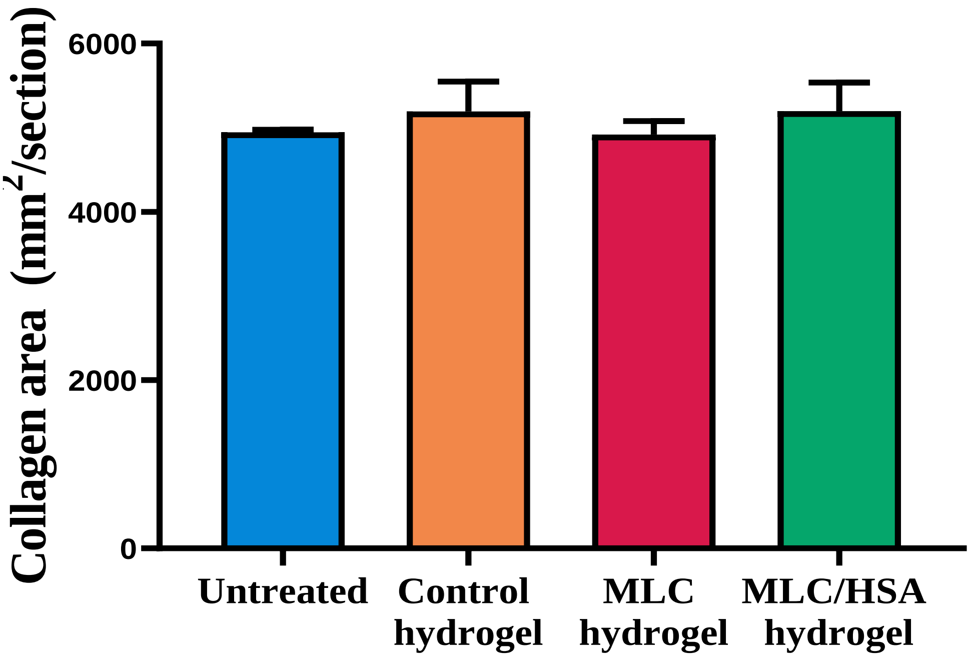

Figure S4. Area of collagen after MT staining in Figure 7B calculated using Teledyne Lumenera Infinity3-1 camera and Infinity Analyze program. No significant difference among groups was observed. Each point represents the mean ± SD (n = 4).
